# Remarks on the Efficacy of the Extract of Hemlock in the Cure of Tetters, and Particularly in the Cure of an Inveterate Disease of the Bladder

**Published:** 1804-04-01

**Authors:** Louis Valentin

**Affiliations:** late Physician of the French Forces in St. Domingo


### Remarks *on the* Efficacy *of the* Extract *of* Hemlock *in the* Cure *of* Tetters, *and particularly in the* Cure *of an inveterate* Disease *of the* Bladder. *By* Louis Valentin, M.D. *late Physician of the French Forces in St. Domingo.*

M. DAVID, an inhabitant of Cape François, 77 years of age, was tormented, for more than three years, by a strangury, for which he had taken the advice of many gentlemen of the faculty, and had used unsuccessfully a great variety of means, both internal and external, and finally bougies were introduced into the urethra. At length he called for my advice, about the latter part of the year 1791; and, amongst the exact accounts of his life, he observed that, twenty-eight years before that period, having been obliged to go to France, on account of an obstinate abdominal dropsy, he abstained entirely from all kinds of drink during twenty-five days on board of the ship, eating only, for his sustenance, some dry food; that when he landed at Bordeaux his belly was very much lessened, and in a short time after the cure was completed without any medicine. Some time after his return to St. Domingo, at the quarter of Dondon, near Cape-François, where his estate was, the dropsy began again to appear; and, in a second voyage, he was thoroughly cured by a methodical treatment under a French physician; but he was very sorry to be obliged to confess that he had abstained from drinking water only fifteen days in his passage. Thereupon he concluded that, being able to do in some degree without substantial food, he was determined to take any advice that I should give him, in order to alleviate the severity of his sufferings.

M. David had almost always lived a regular life, and his blood had never been infected by the venereal disease. His constitution was dry and lean. Having examined his disorder, I saw him making water by drops, with burning pain in the urethra, chiefly in the neck of the bladder; and, during one hour, by reiterated contractions and efforts, he never discharged but one, two, or three spoonfuls of urine. He was afraid of drinking any more, lest it should disturb his rest, of which he had been so long deprived. No obstacle was in the canal, no tumour in the rectum intestinum, nor in the prostate gland. The hypogastric gastric region was nearly in a natural state. A looseness now and then disturbed and weakened him much.

There might be two immediate causes suspected — a stone in the bladder, or a particular disease in the membranes of that organ. Having introduced a sound, and after exploring attentively, I discovered no extraneous body; but I could not turn about my sound in its cavity as in other men. I found every where by the end of the sound a resistance as in a pocket of leather. I thought that a catheter of elastic gum might be useful in order to let out the superabundance of water, which the bladder could not hold without the most acute pain. But it was impossible for the patient to endure the catheter even two hours, notwithstanding his extraordinary courage. The half baths, the emollient topics, and all drink or other lenient medicines whatever, had never relieved him but for a moment.

Amongst my inquiries about remote causes of an old disorder, I learned that he was subject to the erysipelas and tetters, and showing me his left leg, I perceived a great mark of a ring-worm upon the inside of it, which itched so extremely that it caused him to scratch off the skin. Instructed already, by experience, in the effects of the repulsion of some acrid humour from the skin, and even of the gout and rheumatism upon the *viæ urinariæ*, I was induced to believe that his bladder could be affected by nothing but the humour of the ring-worm; that the membranes of it were thickened and hardened, and the size of the cavity decreased considerably in proportion to the length of its pathological state; and therefore the patient could not delay to evacuate a small quantity of urine, the contact of which provoked an irritability already much increased.

I began the cure with two pukes of ipecacuanha root, as much on account of the lax as to excite a shake from the centre to the circumference. Afterwards I ordered the extract of hemlock, increasing every day the dose, with six, eight, or ten grains; soon after it was brought up to a drachm. Then I continued the same weight more than a month, until he got rid of a great propensity to sleep (which he felt every day after dinner), and of some convulsive motions in his lips. The dose was after that increased to three drachms every day. I then perceived that the bladder kept in a great quantity of urine, and its extreme sensibility was much abated. However, an abounding excretion of spittle, with an inspissated mucosity, without any affection, either of the throat, the tonsils, gums, &c. was an inducement to diminish the dose for a short time.

Our patient drank also the juices of some of the depuratory herbs, but the diarrhœa coming on, he forsook them. I ordered to renew the dose, by degrees, to three drachms. He endured it perfecly well; and, in the sight of every body, he evacuated without interruption, and almost without pain, a large tumbler full of urine. He felt that the bladder was more extended, and that he could contain his water a long time. He went from home with a design to take a walk, what he had not done for three years past, being obliged to stand every moment, in order to make a few drops of water.

The ring-worm of the leg and the itching appeared no more. I had fixed a cautery under the knee of the same side, but it never discharged any matter. All the irritating and stimulating topics on the tetter, though in a small quantity, brought their action into the bladder and the canal of the urethra. I forsook them to wash only the leg with the decoction of althea.

In fine, sleep and the other functions of that age (the appetite excepted) were restored, after the use of the hemlock, in one year. In computing exactly, in every particular, the quantity of that extract, we found that M. David had taken sixty-four ounces of it, or four pounds. (There are sixteen ounces in the French pound.)

Some time after this cure, an insurrection happened in the town, in which one party attacked another. M. David being quietly seated down at home, near the door, St. Simon's Street, was, by chance, struck in the breast, and killed immediately, by a gun-shot, which was designed not for him but for a horseman who was escaping. I seized upon this occasion to open the dead body, and I called Dr. Baradat with a design to examine the bladder with me. We found it sound, smaller than in a natural state, and its membranes but a little thicker about the lower part.

### REFLECTIONS *on the* EXTRACT *of* HEMLOCK (*Conium Maculatum.* Linn.)

We see, by the above observation, not only the good effects of this remedy, but also the strong dose which the patient has borne without inconvenience (the inodorous salivation being excepted), when three drachms every day were taken. I have always ordered strong doses of it, and it it never produced that last effect. It might, perhaps, be said, that the extract of hemlock contained mercury; but it is wrong, for no experiments have given any proof on that subject. It was fresh and well chosen, and appeared to be very good, being procured from Messrs. Saussay and Brus, apothecaries at the Cape. The same remedy has never produced that effect in any other instance; having made a copious and successful use of it in the cure of obstinate tetters in the West Indies, some time together, with the strained juices from herbs of a soapy and nitrous quality.

However, I have recently seen at Norfolk, in Virginia, a person to whom I prescribed the extract of hemlock, by degrees to one drachm per diem, casting up a great deal of spittle, afflicted with a sore throat, sore gums, tonsils, &c. Gold, when rubbed with that medicament, would become white immediately, and I discovered easily the quick-silver. It was a mixture of Bellost's pills and a small quantity of extract.

Every physician knows how exceedingly the extract of hemlock has been praised by Dr. Storck for the cure of cancers; but numerous experiments have proved its insufficiency, not to say its inefficaciousness; and its virtues in those cruel diseases are now come to nothing, as well as the praises and boastings of its admirers. It is not the case as to the diseases of the skin, ulcers, and some oppilations issuing from them, and even sometimes from a venereal disease, after an unsatisfactory treatment with mercury. The climbing morel *(solanum scandens seu dulcamara)*, so celebrated by Dr. Carere, of Paris, and lastly by Dr. Otto, from Gotha; the bark of the pyramidal elm, by Dr. Banau; the soap-wort *(saponaria officinalis,)* by Dr. Retz; the antimony of Jacquet; and a great many other chemical compositions, which, nevertheless, are not to be rejected, seem not to produce so good effects as the mere extract of hemlock, especially when its use is combined with a vegetable diet and exercise. All animal and greasy food, fit to increase or maintain the bilious plethora, ought to be interdicted; for the remote causes of those disorders exist undoubtedly in the organs of digestion, which have a great connection with the skin, but essentially in the liver. Therefore Galen says judiciously, "*Herpetes biliosus procreat succus.*"

This remedy must have an excellent effect, taken in sufficient doses, and continued a long time. At St. Domingo I joined with it the cold-bath; I repeated, from time to time, time, vomits with tartar-emetic, preferring often a drink with vinegar, sugar and water, to that of sarsaparilla decoction. Sweats are in those cases always salutary, but they must not be provoked with hot and acrid sudorifics. Among the five kinds of tetters with which we are acquainted, the *herpes pustulosa et squamosa,* and the *herpes viva et suppurans,* are there the most common; and often many people are cured by changing the climate only.

The desiccative and repelling ablutions and liniments are sometimes employed successfully, but it is only about the end of the treatment, and they must be prescribed cautiously.

Permit me to observe, that the alkali volatile fluid, and the sudorific syrups of mercury, which some surgeons of St. Domingo make use of, have frequently produced pernicious effects. Mercury is very noxious when there is not a venereal taint; and those ring-worms do not originate often from it, as is commonly believed.

